# Walther Riese (1890–1976)

**DOI:** 10.1007/s00415-013-7237-z

**Published:** 2014-01-08

**Authors:** Frank W. Stahnisch, Stephen Pow

**Affiliations:** Department of Community Health Sciences and Department of History, Hotchkiss Brain Institute and Institute for Public Health, University of Calgary, 3280 Hospital Drive N.W., Calgary, AB T2N 4Z6 Canada

**Fig. 1:**
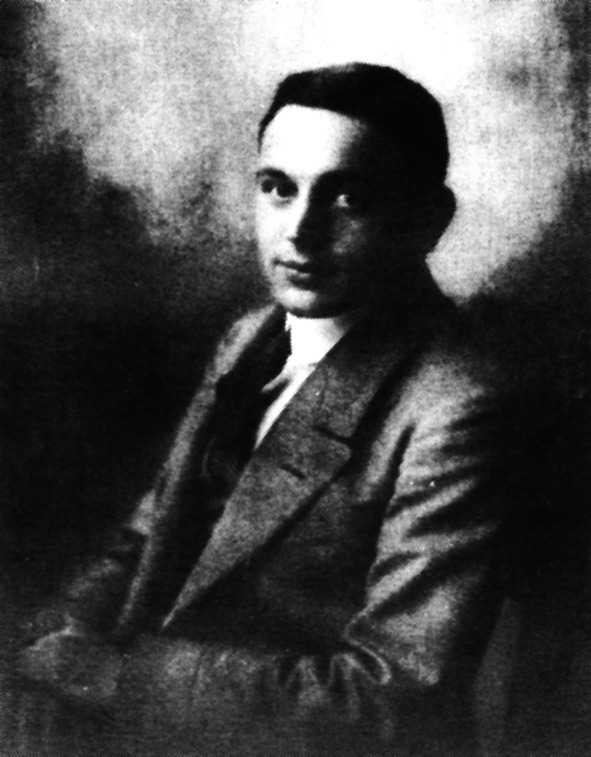
Photograph of Walther Riese (ca. 1914). From: Eiswirth I (1983) Walther Riese (1890–1976)—Leben und Werk, Cologne, Diss Med, p. 7

Owing to advances in the understanding of neurophysiological responses to high-stress situations, post-traumatic stress disorder (PTSD) has become a topic of widespread attention in recent years. While anthropologist Allan Young depicts PTSD as an “invented” product of contemporary psychiatric approaches, this deviates slightly from scholarship that holds that PTSD-like conditions preceded the American Psychiatric Association’s acceptance of the very notion in DSM III (1980) [10]. What appears as a contemporary phenomenon is the intensifying debate surrounding the socio-ethical ramifications of neurological and psychiatric assessments of PTSD within judicial proceedings related to financial compensation claims [4]. However, German neurologist and psychiatrist Walther Riese’s (1890–1976) efforts to assist the claims of veterans suffering from traumatic neurosis (*Unfallneurose*) following World War One suggest that the modern debate is not without antecedents (Fig. 1).

Walther Riese was born as the youngest of eight children to affluent parents in Berlin’s highly assimilated Jewish community and led a sheltered childhood. While Riese demonstrated a logical mind, he showed early aptitude for the humanities that became evident throughout his life. Decades later, a colleague would note that he was equally adept in German, English, and French when he wrote on topics in the history of neurology and neuroethics, while his elaborate style resembled that of the seventeenth-century philosophers he admired [6]. Riese passed his exams at the University of Berlin in 1914, and since physicians were needed for Germany’s war effort, he was soon sent to the University of Koenigsberg, where he earned his doctorate, serving as assistant to psychiatrist Ernst Meyer (1871–1931). It is interesting to note with respect to Riese’s defence of the legitimacy of *Unfallneurose* that he himself suffered nervous exhaustion during his first winter in Koenigsberg, becoming a patient at the same clinic where he had worked with the many war-traumatized soldiers. This was only alleviated by the prolonged visit of his new bride, and later child psychiatrist, Hertha Riese (1892–1981) [3].

Walther Riese’s views, however, owe more to his research at the University of Frankfurt’s *Institute for Research into the Long-term Effects of Brain Injuries* (1917–1933). Here he was brought into what would prove a lifelong collaboration with neurologist Kurt Goldstein (1878–1965) whose clinical observations of thousands of patients led him to a holistic view of neurological function, which Riese came to share [5]. Riese’s later *Principles of Neurology in Light of their Present Use* (1950) further acknowledged Swiss neurologist Constantin von Monakow’s (1853–1930) related work as a powerful influence.

World War One had left approximately 300,000 German veterans diagnosed by neurologists and psychiatrists as suffering from war neurosis. However, in the atmosphere of financial collapse that characterized Weimar Germany, much hostility from state administrators greeted the returning “pension neurotics” who were seen as a burden to an overtaxed welfare system [2]. Correspondingly, neurologists and psychiatrists providing assessments (*Klinikgutachten*) to the National Insurance System decided frequently in favour of the insurers’ interests. Riese’s rejection of this practice found expression in the publication of *Die Unfallneurose* (1929), which likewise is an early document of his concerns with neuroethical problems:

“The problem of the *Unfallneurose* amounts to a problem only in so far as it relates to the critical and decisive interference of the physician with the patient.” [7].

Riese’s career in Germany ended with the Nazi takeover in January of 1933. Walther, as a Jewish physician, and Hertha, who headed a socialist ambulatory and birth control clinic in Frankfurt, immediately found themselves in prison. The particular reason provided for their arrest was the book *Die Unfallneurose*, which had so roundly denounced the conduct of state authorities, earning the couple many powerful enemies [3]. They and their two daughters only narrowly escaped to Switzerland in March, before continuing to France with the help of a Rockefeller research fellowship. Here, Riese re-established a research program on comparative neuroanatomy at the University of Lyon, until the conquest of France by the *Wehrmacht* (1940) again put their lives in danger. They managed to escape through Vichy France to Rabat, Morocco when another Rockefeller fellowship allowed them to reach the United States [9].

Supported by letters of reference from his mentor Goldstein, who had emigrated to New York in 1935, Riese found a reliable home in the Virginia Medical College in Richmond, where he first served as a neurological researcher and later in the capacity of a professor of neuroanatomy and history of medicine (1941–1976). He continued to write prodigiously, though his publications moved further and further away from Goldstein’s “holistic neurology.” Between 1959 and 1975, Riese concentrated on courses with history of medicine and neuroethical content, which also found their way into the fellowship program of the American Academy of Neurology and panels at the American Association for the History of Medicine. Some of Riese’s more notable works are *The Conception of Disease* (1955) and *A History of Neurology* (1959). In his philosophical views, Riese now advocated for ethical considerations based on a deep analysis of history and its present uses, the alignment of medical decision-making with clinical experiences, along with the teaching of neuroethical content to medical students and practitioners—all at a time when the term of “*neuroethics*” had not even appeared in the literature [8].

In 1969, near the end of his life, Riese’s pioneering work in traumatology, neurohistory and neuroethics was also recognized in his former homeland. As part of the process of redress for Nazi crimes (*Wiedergutmachung*), Riese finally received an honorary title of Professor Emeritus at Frankfurt University, where he had so extensively worked during the interwar period [1].

We are grateful for support from the Mackie Family Collection in the History of Neuroscience, the Hotchkiss Brain Institute, the Institute for Public Health (all: Calgary), the Rockefeller Archive Center (New York), and an Open Operating Grant (no/EOG-123690) from the Canadian Institutes of Health Research. We graciously thank Dr. Darwin Stapleton (Rockefeller Archive Center) and Dr. Paul Weindling (Oxford Centre for the History of Medicine) for their assistance.
